# Correlation between Imaging Markers Derived from PET/MRI and Invasive Acquired Biomarkers in Newly Diagnosed Breast Cancer

**DOI:** 10.3390/cancers15061651

**Published:** 2023-03-08

**Authors:** Kai Jannusch, Ann-Kathrin Bittner, Nils Martin Bruckmann, Janna Morawitz, Cleo Stieglitz, Frederic Dietzel, Harald H. Quick, Hideo A. Baba, Ken Herrmann, Lale Umutlu, Gerald Antoch, Julian Kirchner, Sabine Kasimir-Bauer, Oliver Hoffmann

**Affiliations:** 1 Department of Diagnostic and Interventional Radiology, University Dusseldorf, Medical Faculty, 40225 Dusseldorf, Germany; 2 Department Gynecology and Obstetrics, University Hospital Essen, University of Duisburg-Essen, 45147 Essen, Germany; 3 High-Field and Hybrid MR Imaging, University Hospital Essen, University Duisburg-Essen, 45147 Essen, Germany; 4 Erwin L. Hahn Institute for Magnetic Resonance Imaging, University Duisburg-Essen, 45141 Essen, Germany; 5 Institute of Pathology, University Hospital Essen, University of Duisburg-Essen, 45147 Essen, Germany; 6 Department of Nuclear Medicine, University Hospital Essen, University of Duisburg-Essen, 45147 Essen, Germany; 7 Department of Diagnostic and Interventional Radiology and Neuroradiology, University Hospital Essen, University of Duisburg-Essen, 45147 Essen, Germany

**Keywords:** PET/MRI, breast cancer, imaging biopsy, DTC

## Abstract

**Simple Summary:**

Histological and molecular breast cancer (BC) characteristics are highly important for individualized therapeutical strategies and personalized risk assessment. Despite the improvement of existing image-based staging examinations over the last years, especially the implementation of PET/MRI examinations at leading tumor centers, the invasive part of BC staging is unavoidable. However, the diagnostic potential of PET/MRI has not yet been fully explored. Thus, this study aimed to analyze possible correlations between PET/MRI imaging markers and invasive acquired biomarkers in newly diagnosed early BC to possibly spare invasive procedures for BC patients in the future. At a population of 169 women a correlation between glucose metabolism and estrogen-receptor and progesterone-receptor expression, Ki67, and tumor grading was shown, whereas no correlation regarding disseminated tumor cells (DTCs) could be found. Thus, [^18^F]FDG-PET/MRI may give a first impression of BC-receptor status and BC-tumor biology during initial staging by measuring glucose metabolism but cannot distinguish between DTC-positive/-negative patients and replace biopsy.

**Abstract:**

Purpose: Evaluate the diagnostic potential of [^18^F]FDG-PET/MRI data compared with invasive acquired biomarkers in newly diagnosed early breast cancer (BC). Methods: Altogether 169 women with newly diagnosed BC were included. All underwent a breast- and whole-body [^18^F]FDG-PET/MRI for initial staging. A tumor-adapted volume of interest was placed in the primaries and defined bone regions on each standard uptake value (SUV)/apparent diffusion coefficient (ADC) dataset. Immunohistochemical markers, molecular subtype, tumor grading, and disseminated tumor cells (DTCs) of each patient were assessed after ultrasound-guided biopsy of the primaries and bone marrow (BM) aspiration. Correlation analysis and group comparisons were assessed. Results: A significant inverse correlation of estrogen-receptor (ER) expression and progesterone-receptor (PR) expression towards SUV_max_ was found (ER: *r* = 0.27, *p <* 0.01; PR: *r* = 0.19, *p <* 0.05). HER2-receptor expression showed no significant correlation towards SUV and ADC values. A significant positive correlation between Ki67 and SUVmax and SUV_mean_ (*r* = 0.42 *p <* 0.01; *r* = 0.19 *p <* 0.05) was shown. Tumor grading significantly correlated with SUV_max_ and SUV_mean_ (ρ = 0.36 and ρ = 0.39, both *p*’s < 0.01). There were no group differences between SUV/ADC values of DTC-positive/-negative patients. Conclusions: [^18^F]FDG-PET/MRI may give a first impression of BC-receptor status and BC-tumor biology during initial staging by measuring glucose metabolism but cannot distinguish between DTC-positive/-negative patients and replace biopsy.

## 1. Introduction

Worldwide breast cancer (BC) is the most common cancer in women with almost 2.3 million new diagnoses in 2020 [1,2]. Although the 5-year overall survival (OS) rate is about 90% [3] due to improvement in diagnostics and therapy, a considerable number of patients (approximately 20%) with early BC will develop recurrence, depending on the tumor subtype, etc. [4,5]. For personalized clinical risk assessment and developing a therapeutical strategy, accurate pretherapeutic staging is of particular importance after initial diagnosis in early BC. Implementing positron emission tomography/computed tomography (PET/CT) in BC staging improves diagnostics from stage IIb and higher with a focus on distant lymph node metastases providing a higher accuracy compared to single CT staging [6,7]. Furthermore, the PET component improves the accuracy and seems to have an additive predictive potential for disease recurrence in BC and non-BC studies [8,9,10]. Additionally, PET/magnetic resonance imaging (MRI) is an increasingly important element for BC patients of initial whole-body diagnostics in leading tumor centers and outperforms the staging algorithm currently recommended in BC guidelines as well as PET/CT with a focus on local BC classification and identification of distant metastases [11,12,13,14,15,16,17,18]. This is mainly due to the excellent soft tissue contrast in combination with the obtained multiparametric dataset that allows further tumor classification as well as individual therapeutical strategies [19,20,21]. Furthermore, PET/MRI has the ability to provide functional data related to tumor biology (e.g., cell replication) by using diffusion-weighted imaging and tumor neoangiogenesis by examining dynamic contrast-enhanced MRI sequences [22]. Invasive diagnostics to determine histopathological and molecular characteristics (e.g., molecular subtypes) of BC tumor cells have become highly important for individualized BC therapies and are unavoidable by now [23,24,25]. This applies to diagnostics of early micrometastatic tumor spread to the blood and bone marrow (BM), called minimal residual disease (MRD) [26,27], surviving in a state of dormancy, preferentially in the BM as disseminated tumor cells (DTCs) [28]. DTCs are independent prognostic markers for BC OS, disease-free survival (DFS), and distant disease-free survival (DDFS) in multivariate analysis, undetectable by standard staging methods [29].

The diagnostic potential of PET/MRI on a molecular level has not yet been fully explored. Some studies could already highlight the potential of glucose metabolism of different tumor entities (e.g., gliomas, endometrial cancer, lung cancer, BC) measured by SUV at PET data for the determination of tumor aggressiveness, tumor grading, and tumor subtypes [30,31,32,33,34]. Especially significant correlations of BC glucose metabolism towards histological characteristics of BC regarding estrogen (ER, inverse correlation) and progesterone receptor state (PR, inverse correlation) and tumor aggressiveness (Ki67, verse correlation) could be visualized [35,36]. Furthermore, diffusion-weighted imaging (DWI) of MRI data sets achieved during MRI and PET/MRI delivers information about tumor cellularity and helps to distinguish between cancerous and non-cancerous lesions in BC and non-BC studies [37,38,39,40]. Additionally, Catalano et al. (2017) found a significant inverse correlation between ADC_mean_ measurements from PET/MRI towards human epidermal growth factor receptor 2 (HER2) positivity in invasive ductal BC [35]. To the best of our knowledge, there are currently no studies in the actual literature that evaluate the detection of DTCs using image morphological parameters derived from PET/MRI or other radiological imaging modalities.

Summing up, the individual characterization of BC tumor cells and treatment of BC is substantially and currently an interplay of invasive and imaging diagnostics. In order to deepen the understanding of BC tissue on a molecular level and to supplement the required invasive diagnostics in the future, this study should analyze possible correlations between imaging markers derived from PET/MRI and invasive acquired markers/tumor biology in newly diagnosed early BC on a clinically feasible basis.

## 2. Materials and Methods

### 2.1. Patients

The institutional review boards of the University Duisburg-Essen, Germany (study number 17-7396-BO) and Düsseldorf, Germany (study number 6040R) approved this study and it was performed in conformance with the Declaration of Helsinki [41]. Data acquisition was performed between March 2018 and December 2021. Women with newly diagnosed, therapy-naive early BC were included in this retrospective trial and all patients met the following inclusion criteria: (i) tumors presenting with at least cT2 tumor stage; (ii) triple-negative BC (TNBC) of any tumor size; or (iii) tumors with intermediate risk due to clinicopathological parameters, without having performed multigene assays at that point or high risk, presenting with at least one of the following characteristics: >cT1c, Ki67 > 14%, HER2 overexpression, G3. The following exclusivity criteria have been defined: (i) former malignancies within the last five years, (ii) contraindications to MRI or MRI contrast agents, and (iii) pregnancy or breastfeeding. Written informed consent form was obtained from all patients. All patients underwent a dedicated breast [^18^F]FDG-PET/MRI for initial staging purposes.

### 2.2. PET/MRI

The dedicated [^18^F]FDG-PET/MRI examinations were performed on an integrated 3-Tesla PET/MRI system (Biograph mMR, Siemens Healthcare GmbH, Erlangen, Germany). The average delay was 69 ± 15 min after injection of bodyweight-adapted dosage of [^18^F]-FDG (4 MBq/kg bodyweight). To ensure blood glucose levels of below 150 mg/dL, blood samples were obtained and patients need to fast six hours prior to injection.

The first examination was a dedicated and comprehensive breast-[^18^F]FDG-PET/MRI examination, performed in a head-first prone position utilizing a dedicated 16-channel radiofrequency (RF) breast coil (Rapid Biomedical, Rimpar, Germany), developed and designed for use in integrated whole-body PET/MR imaging [42]. PET data and MRI data of both breasts were acquired simultaneously with an acquisition time of 20 min per bed position. PET image reconstruction was performed subsequently using an iterative ordered subset expectation maximization algorithm, 3 iterations and 21 subsets, a Gaussian filter with 4 mm full width at half maximum, and a 256 × 256 image matrix. PET data of the patient tissues were automatically attenuation corrected using an implemented four-compartment model attenuation map (μ-map) calculated from fat-only and water-only data sets, as obtained by Dixon-based sequences. The attenuation correction for the 16-channel RF breast coil was automatically performed by the PET/MRI system. A 3D attenuation template of the RF breast coil based on CT data was implemented into the PET data reconstruction process for this purpose [42].

The dedicated breast MRI protocol comprised the following sequences:

(i) A transversal T2-weighted (T2w) turbo-spin echo (TSE) fat-saturated sequence with a slice thickness of 7 mm (TE 97 ms; TR 2840 ms; FOV 400 mm; phase FOV 75%; acquisition matrix 256 × 192, in-plane resolution 1.6 × 1.6 mm^2^)
(ii) A transversal diffusion-weighted echo-planar imaging (EPI) sequence with a slice thickness of 5.0 mm (TR 8000 ms; TE 81 ms; b-values: 0, 400 and 800 s/mm^2^, matrix size 192 × 156; FOV 420 mm, phase FOV, 81.3%; GRAPPA, acceleration factor 2; in-plane resolution 2.2 × 2.2 mm^2^)
(iii) Six repetitions of a transversal 3-dimensional fast low-angle shot (FLASH) T1w sequence with a slice thickness of 7 mm (TE 3.62 ms; TR 185 ms; FOV 400 mm; phase FOV 75%; acquisition matrix 320 × 240, in-plane resolution 1.3 × 1.3 mm^2^) for dynamic contrast-enhanced imaging. A dose of 0.2 mmol/kg bodyweight gadoterate meglumine (Dotarem, Guerbet, Sulzbach, Germany) was injected intravenously after the first FLASH sequence with a flow of 2 mL/s using an automated injector (Spectris Solaris, MR Injection System; Medrad, Pittsburg, PA, USA). Subsequent automated image subtraction was performed.

Subsequent to the dedicated breast [^18^F]FDG-PET/MRI examination patients were positioned head-first-supine for a second whole-body [^18^F]FDG-PET/MRI examination. Data acquisition was performed in 4–5 bed positions with an acquisition time of three min per bed position. The following vendor-provided RF coils for MR signal reception that are part of the PET/MR system were used: (i) 16-channel head/neck RF coil (ii) 24-channel spine-array RF coil (iii) 3–4 flexible body array RF coils each with six channels for whole-body coverage (head to mid-thigh). Both the head/neck and spine-array RF coil are included in the automated attenuation correction procedure of the PET/MRI system [43]. The flexible-body array RF coils are designed as PET-transparent as possible and are not considered in the attenuation correction procedure [44,45].

All PET images were reconstructed using the iterative ordered-subset expectation maximization (OSEM) algorithm, three iterations, 21 subsets, a Gaussian filter with 4 mm full width at half maximum (FWHM), and a 344 × 344 image matrix. For MR-based attenuation correction of the patient tissues, a two-point (fat, water) coronal 3D-Dixon-VIBE sequence was acquired to generate a four-compartment model (background air, lungs, fat, muscle).

The dedicated whole-body [^18^F]FDG-PET/MRI examination comprised the following sequences:

(i) A transverse T2-w half Fourier acquisition single-shot turbo spin echo (HASTE) sequence in breath-hold technique with a slice thickness of 7 mm (TE 97 ms; TR 1500 ms; turbo factor (TF) 194; FOV 400 mm; phase FOV 75%; acquisition matrix 320 × 240 mm; in-plane resolution 1.3 × 1.3 mm^2^; TA 0:47 min/bed position)
(ii) A transversal diffusion-weighted (DWI) echo-planar imaging (EPI) sequence in free breathing with a slice thickness of 5.0 mm (TR 7400 ms; TE 72 ms; b-values: 0, 500 and 1000 s/mm^2^, matrix size 160 × 90; FOV 400, phase FOV, 75%; GRAPPA, acceleration factor 2; in-plane resolution 2.6 × 2.6 mm^2^; TA 2:06 min/bed position)
(iii) A fat-saturated post-contrast transverse 3-dimensional volumetric interpolated breath-hold examination (VIBE) sequence with a slice thickness of 3 mm (TE, 1.53 ms; TR, 3.64 ms; flip angle 9°; FOV 400; phase FOV 75%; acquisition matrix 512 × 384, in-plane resolution 0.7 × 0.7 mm^2^; TA 0:19 min/bed position)

### 2.3. Image Analysis

All images were analyzed using a dedicated OsiriX workstation (Version 9.0.2; Pixmeo SARL, Bernex, Switzerland). A breast-imaging specialist with more than 10 years of experience and a hybrid-imaging specialist with two years of experience performed the data evaluation.

**Figure 1:**
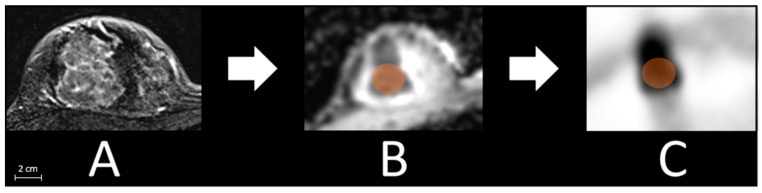
Exemplified measurement processes with detecting the lesion in T1 weighted post-contrast sequence (**A**), pacing a tumor size adapted volume of interest (VOI, orange, 2.3 cm here) in the ADC map (**B**), and afterward copying the VOI to the corresponding slice of the PET images (**C**).

**Breast lesion:** After defining the malign breast lesion at T1 weighted post-contrast sequence, a tumor size adapted volume of interest (VOI) that adequately captured the breast cancer lesion was set in the ADC map of each patient. Afterward, the VOI was copied to the corresponding PET images to match the identical plane and position. Finally, in both images, a manual reshape was performed to avoid pixel loss (Figure 1).

**Figure 2:**
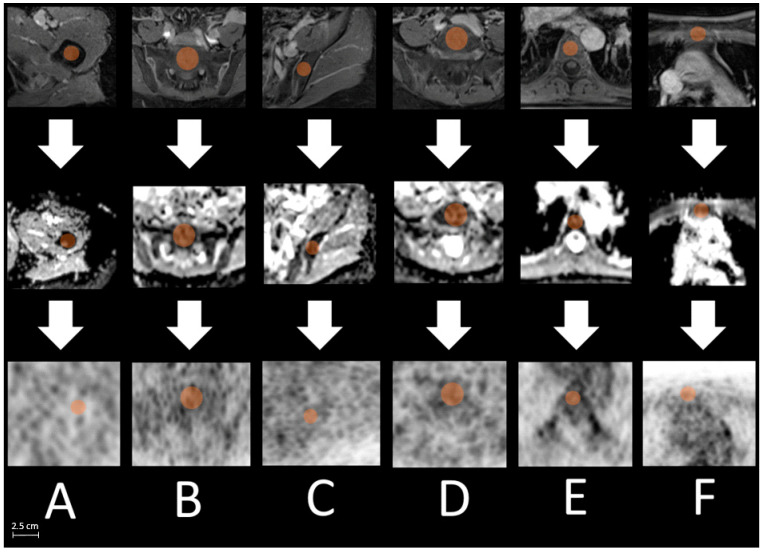
Exemplified measurement processes with locating defined bone regions ((**A**): right femur, (**B**): os sacrum, (**C**): os ilium, (**D**): L 5, (**E**): T 7, (**F**): sternum) in T1 weighted post-contrast sequence ((**upper**) row), pacing a bone size adapted volume of interest (VOI, orange, max. 2.5 cm os sacrum) centrally in each bone location in the ADC map ((**middle**) row) and afterward copying the VOI to the corresponding slice of the PET images ((**lower**) row).

**Bone marrow:** Matching with transverse T1-weighted post-contrast sequence, a bone size adapted VOI (on average 1.0 cm spherical diameter) was placed in the ADC map centrally in each of the following bones: femur right, os sacrum, os ilium right, lumbar vertebral body 5 (L 5), thoracic vertebral body 7 (T 7), and sternum. Afterward, the VOI was copied to the corresponding PET images to match the identical plane and position. Finally, in both images, a manual reshape was performed to avoid pixel loss (Figure 2).

OsiriX automatically calculated SUV_max_ and SUV_mean_ from PET, and ADCmean from DWI of each PET/MRI data set. In partly included pixels, the software used subpixel interpolation.

### 2.4. Histopathological Examination

For each patient, tumor grading (G1–G3), type, and tumor biology including estrogen (ER) and progesterone receptor (PR), as well as human epidermal growth factor receptor 2 (HER2) status and Ki67 (proliferation marker) were assessed according to World Health Organization classification after ultrasound-guided biopsy. Tumor biology as well as tumor subtypes are essential for therapeutical strategies [46]. Patients were divided into subgroups: Luminal-A-like, Luminal-B-like HER2 negative, TNBC, and HER2 positive [47,48].

### 2.5. Selection and Detection of Disseminated Tumor Cells (DTCs)

**Figure 3:**
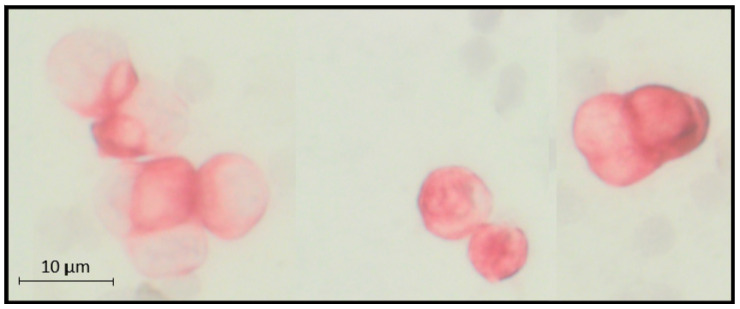
Example of microscopic visualized cytokeratin-positive disseminated tumor cells (on average 11 µm) in bone marrow aspirate from the anterior iliac crests.

Between 10 and 20 mL BM was aspirated from the anterior iliac crests of all patients at the beginning of surgery of the primary tumor, before the start of any therapy, and processed within 24 h. DTC isolation and detection were performed on the basis of the recommendations for standardized tumor cell detection, published by the German consensus group of Senology [49]. Details of the staining procedure, for example, the number of evaluated slides, controls, and cell detection, have been described elsewhere [50]. Briefly, BM cells were isolated from heparinized BM (5000 U/mL bone marrow) by Ficoll–Hypaque density gradient centrifugation (density 1.077 g/mol; Pharmacia) at 400× *g* for 30 min. Slides were analyzed for DTCs by immunocytochemistry using the pan-cytokeratin anti-body A45-B/B3 (see Figure 3). Microscopic evaluation of the slides was carried out using the ARIOL system (Applied Imaging, San José, CA, USA), according to the ISHAGE evaluation criteria [51].

### 2.6. Statistical Analysis

Statistical analysis was performed using SPSS Statistics 26 (IBM Corp, Chicago, IL, USA). Pearson’s (ER, PR, Ki67) and Spearman´s (HER2/neu, molecular subtype, tumor grading) correlation coefficients were calculated to correlate SUV_max_/SUV_mean_ and ADC_mean_ tumor values and immunohistochemical markers or tumor grading. The Mann–Whitney-U-Test was used for group comparison of SUV/ADC values of DTC-positive or -negative patients at defined bone regions. A *p*-value less than 0.05 was considered statistically significant. Data are presented as mean ± standard deviation.

## 3. Results

### 3.1. Patient Population and Histopathological Findings

A total of 169 female patients (56 ± 12 years) were included in this retrospective trial. For the distribution of the cohort into molecular subtypes and tumor grading, see Table 1.

### 3.2. Correlation of Breast Cancer SUV and ADC with Histopathological Breast Cancer Parameters

**Figure 4:**
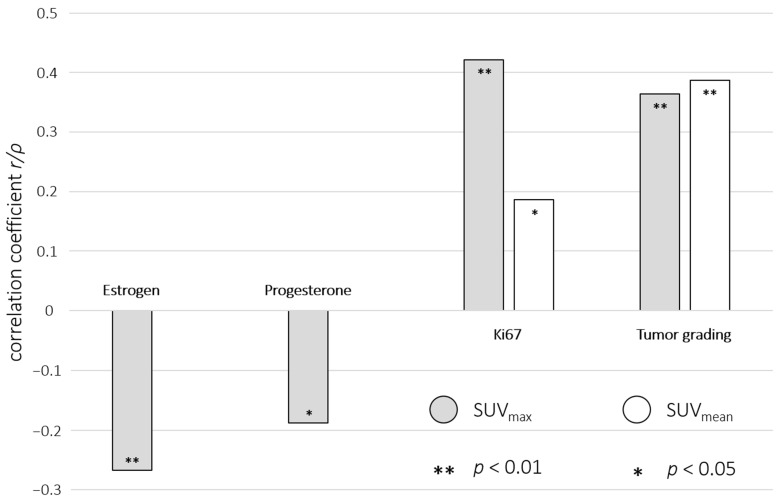
Visualized significant correlations between immunohistochemical markers, tumor grading, and SUV_max_ (grey)/SUV_mean_ (white) from [^18^F]FDG-PET/MRI in combination with their correlation coefficients (*r/*ρ) and *p* values (** *p* < 0.01; * *p* < 0.05).

ER expression was found in 124/169 (73%) patients with a mean expression of 83 ± 29% and a significant inverse correlation with SUV_max_ (*r* = 0.27 and *p* < 0.01). No significant correlation between ER and SUV_mean_/ADC could be found. PR expression was detected in 115/169 (68%) patients with a mean expression of 58 ± 37% and a significant inverse SUV_max_ correlation (*r* = 0.19 and *p* < 0.05). No significant correlation between PR and SUV_mean_/ADC was found. HER2 receptor expression was described in 97/169 (57%) patients, showing no significant correlations with SUV and ADC values. The mean Ki67 was 43 ± 27% and showed a significant positive correlation with SUV_max_ and SUV_mean_ (*r* = 0.42 *p* < 0.01; *r* = 0.19, *p* < 0.05). No significant correlation between the Ki67 index and ADC was found, as well as between molecular subtypes with SUV/ADC. A significant positive correlation of tumor grading with SUV_max_ and SUV_mean_ could be shown (ρ = 0.36 and ρ = 0.39, both *p*’s < 0.01), whereas no significant correlation between tumor grading and ADC was found (Table 2 and Figure 4).

### 3.3. Group Comparison of Bone Marrow SUV/ADC between DTC-Positive and DTC-Negative Patients

In a subgroup of 136 patients, BM was evaluated for DTCs. Due to image artifacts (e.g., after artificial hip implantation), 13 of those patients were excluded from further analysis respective to bone regions (n = 5 right femur, n = 2 os sacrum, n = 2 right os ilium, n = 1 L 5, n = 3 sternum).

**Figure 5:**
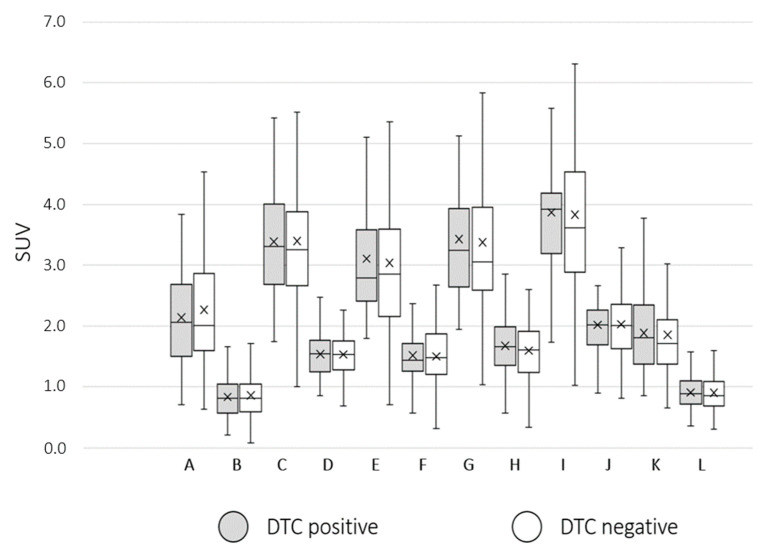
Group comparison between DTC (disseminated tumor cells)-positive (grey box) and DTC-negative (white box) patients towards measured SUV bone marrow values: (**A**) femur right-SUV_max_, (**B**) right femur SUV_mean_, (**C**) os sacrum-SUV_max_, (**D**) os sacrum-SUV_mean_, (**E**) right os ilium SUV_max_, (**F**) right os ilium SUV_mean_, (**G**) L 5-SUV_max_, (**H**) L 5-SUV_mean_, (**I**) T 7-SUV_max_, (**J**) T 7-SUV_mean_, (**K**) sternum-SUV_max_, and (**L**) sternum-SUV_mean_. The crosses indicate the mean values. No significant differences in group comparison were visible.

**Figure 6:**
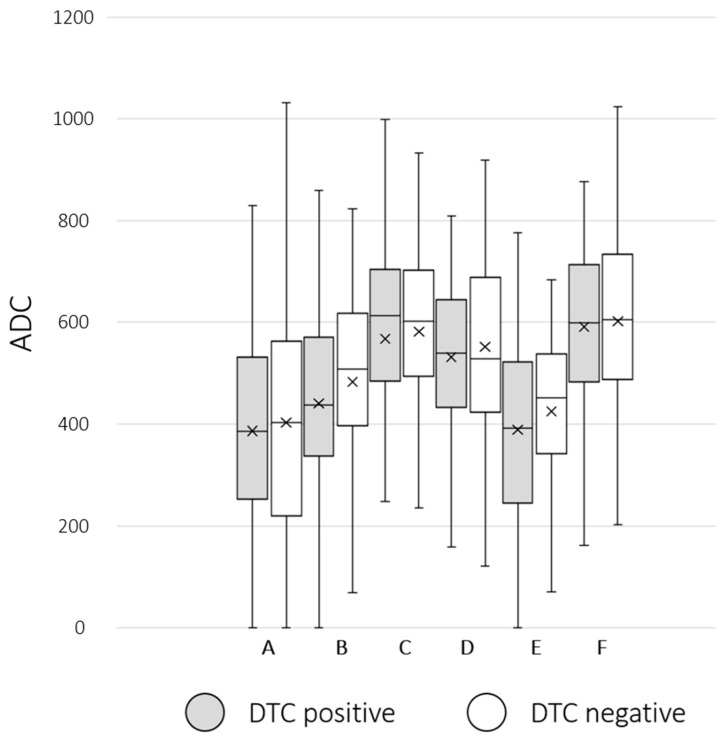
Group comparison between DTC (disseminated tumor cells)-positive (grey box) and DTC-negative (white box) patients regarding ADC_mean_ bone marrow values: (**A**) right femur ADC_mean_, (**B**) os sacrum-ADC_mean_, (**C**) right os ilium ADC_mean_, (**D**) L 5-ADC_mean_, (**E**) T 7-ADC_mean_, and (**F**) sternum-ADC_mean_. The crosses indicate the mean value. No significant differences in group comparison were visible.

The Mann–Whitney-U-Test revealed no significant differences of SUV/ADC values derived from [^18^F]FDG-PET/MRI between DTC-positive and DTC-negative patients concerning defined BM regions: right femur, os sacrum, right os ilium, L 5, T 7, and sternum. For detailed data visualization, see Table 3 and Figure 5 and Figure 6.

## 4. Discussion

Histological as well as molecular BC characteristics are highly important for individualized therapeutical strategies and personalized risk assessment [23]. Despite the improvement of existing image-based staging examinations over the last years, especially the implementation of PET/MRI examinations at leading tumor centers, the invasive part of BC staging is unavoidable. Thus, a biopsy of the primary breast lesion for immunohistochemical data is state-of-the-art. BM aspiration has been performed in many clinics several years ago; however, nowadays, due to time-consuming and cost-effective issues, only a very few still perform it to evaluate DTCs. Nevertheless, the informative potential of hybrid examinations such as PET/MRI is not yet exhausted.

To better understand the influence of histopathological and molecular BC characteristics on imaging markers derived from [^18^F]FDG-PET/MRI, this study aimed to analyze the possible correlations between PET/MRI imaging markers and invasive acquired biomarkers in newly diagnosed early BC patients on a clinically feasible basis. Thus, it could improve personalized clinical risk assessment and the development of therapeutical strategies.

Concordant with Catalano et al. (2017), we found an inverse correlation between SUV_max_ of the malign BC lesion and both ER and PR expression [35]. Thus, a higher SUV_max_ might indicate a lower ER and PR expression. This might be helpful for personalized therapy planning as it is known that information about receptor expression is one necessary component. According to our data information about HER2, expression was not reflected by image morphological data of [^18^F]FDG-PET/MRI. Ki67 and tumor grading positively correlated with SUV_max_ and SUV_mean_ of the malign BC lesion. Similar supporting results were presented by other BC and non-BC studies [36,52,53,54,55,56]. The amount of Ki67 as well as tumor grading indicate the aggressiveness of BC lesions [57,58]. Consequently, the detected positive correlation of both parameters towards SUV values derived from [^18^F]FDG-PET/MRI could give an impression of the tumor aggressiveness during initial staging by measuring SUV values. In patients with more than one malignant breast lesion, this correlation provides the opportunity, if not already performed at the initial puncture, to specifically puncture the breast lesion suspected to have the highest malignant potential at [^18^F]FDG-PET/MRI. Furthermore, Afkari et al. (2021) could show a positive correlation between Ki67 and the risk of bone metastases [59]. As a fast and easily achievable imaging parameter during staging, this might help to improve the staging process giving the investigator the hint that a close look to exclude bone metastases is particularly necessary here [59].

Contrary to Koo et al. (2016), based on our data, there were no significant correlations between molecular subtypes and SUV/ADC values [60]. One potential explanation could be the intermediate- to high-risk population of our cohort including only a small number of patients with Luminal A (4% vs. 60%) tumors and a high amount of HER2-positive (53% vs. 11%) cases [60]. Thus, a group differentiation based on SUV/ADC might be difficult here. Furthermore, there were no correlations of immunohistochemical markers, molecular subtype, and tumor grading toward ADC_mean_ values, which supports the result of some other studies in the current literature [34,35,61,62].

DTCs, detected in BM in about 30% of BC patients, are an independent prognostic marker for disease outcome [27,29]. One therapeutic approach showing an improvement in OS is the application of bisphosphonates [63,64]. Besides reducing skeletal complications, clodronate (clodronic acid) has shown a significant reduction of recurrence to the bone as well as visceral metastasis [65,66,67,68]. Moreover, in a small pilot study, we demonstrated a positive effect of ibandronate treatment on the eradication of DTCs, still present 2–10 years after primary diagnosis [68] which was shown to successfully improve outcomes in primary as well as locally advanced BC patients receiving adjuvant/neoadjuvant chemotherapy [50,67]. Unfortunately, DTCs are undetectable by standard staging methods and BM aspiration is needed [69]. Changes in [^18^F]FDG-PET/MRI imaging markers (SUV/ADC) of the BM in case of DTC positivity would help to spare patients a BM biopsy. According to our data, there is no hint that the existence of DTCs changes [^18^F]FDG-PET/MRI imaging markers. The results fit well with the underlying pathophysiology of the disease [70]. As described previously, DTCs are the result of a micrometastatic tumor spread; however, we here only can describe the presence of cytokeratin-positive cells without any further characterization of the cells. Although little is known about the survival conditions of DTCs in the BM, some DTCs have been shown to have stem cell characteristics with the ability of self-renewal [71,72], and we recently demonstrated that early-stage-diagnosed BC patients harboring DTCs expressing the chemokine receptor type 4 (CXCR4) and the transcription factor JUNB had a higher risk for relapse [73]. Furthermore, DTCs have to adapt to new environmental conditions. In this regard, a subpopulation of osteoblasts was identified that was manipulated in their function by DTCs, so-called educated osteoblasts, which in turn crosstalk with DTCs via proteins and soluble factors leading to a reduction in BC cell proliferation and metastatic latency [74]. Dormant DTCs would probably not cause any significant metabolic activity in these cells, which is essential for the accumulation of [^18^F]FDG. However, only a comprehensive characterization of DTCs, not feasible in daily clinical routines, could finally answer that question. Moreover, DTCs are rarely building larger cell clusters. Thus, the missing metabolic activity and the distribution of DTCs make it actually hard to visualize bone marrow SUV and ADC changes at highly accurate [^18^F]FDG-PET/MRI staging in a clinical implementable setting.

As a one-stop examination, [^18^F]FDG-PET/MRI has a significant advantage for BC patients [14,15,16]. Switching between different modalities and scheduling different appointments during guideline-compliant staging is reduced by implementing PET/MRI as a staging method of choice in leading tumor centers. A further major strength of hybrid imaging is the ability to acquire a multiparametric data set. In particular, since the limitations of this examination have not yet been finally clarified, it would be of great advantage for BC patients to identify histopathological characteristics of the tumor within the imaging process (imaging biopsy).

The visualized [^18^F]PET association with receptor status and histopathological parameters might help predict BC types and identify BC lesions with the highest malignant potential and thus improves personalized therapy planning with guide-targeted therapies. Nonetheless, [^18^F]FDG-PET/MRI might find one of its limitations in replacing the BM aspiration to evaluate the DTC status of the patients.

This study has some limitations. First, we only evaluated 136/169 patients’ data regarding BM. Nevertheless, this is the first study that evaluates if [^18^F]FDG-PET/MRI can replace a BM aspiration for the detection of DTCs. Furthermore, histopathological sampling was derived from core needle biopsy; therefore, the biopsy did eventually not represent the whole lesion, which is a well-known problem of other BC studies.

## 5. Conclusions

[^18^F]FDG-PET/MRI may give a first impression of BC-receptor status and tumor biology during initial staging by measuring glucose metabolism but cannot distinguish between DTC-positive/-negative patients. Thus, regarding multicentricity, it could improve personalized therapy planning with guide-targeted therapies but cannot replace biopsy.

## Figures and Tables

**Table 1:** Distribution of the cohort (n = 169) into (A) molecular subtypes and (B) tumor grading.

**A**	**Molecular** **Subtypes**	**Luminal A**	**Luminal B** **HER2−**	**Triple** **Negative**	**HER2+**
	total 169	7 (4%)	42 (25%)	23 (14%)	97 (57%)
**B**	**Tumor Grading**	**Grade 1**	**Grade 2**	**Grade 3**	
	total 169	3 (2%)	100 (59%)	66 (39%)	

**Table 2:** Correlation between immunohistochemical markers (estrogen, progesterone, HER2, Ki67), molecular subtype, tumor grading in relation to SUV, and ADC measurements from [^18^F]FDG-PET/MRI.

	Malign Breast Lesion PET/MRI		
	SUV_max_	SUV_mean_	ADC_mean_
**Estrogen**	*r* = **−0.27** **	*r* = −0.07	*r* = −0.06
**Progesterone**	*r* = **−0.19** *	*r* = −0.11	*r* = −0.15
**HER2/neu**	ρ = −0.07	ρ = −0.04	ρ = −0.06
**Ki67**	*r* = **0.42** **	*r* = **0.19** *	*r* = −0.08
**Molecular subtype**	ρ = 0.04	ρ = 0.06	ρ = −0.01
**Tumor grading**	ρ = **0.36** **	ρ = **0.39** **	ρ = 0.02

* Bold values indicate significance at *p* < 0.05/** Bold values indicate significance at *p* < 0.01.

**Table 3:** Comparison of SUV_max_/SUV_mean_/ADC_mean_ values derived from defined BM regions at [^18^F]FDG-PET/MRI between DTC-positive and DTC-negative patients using Mann–Whitney-U test. Descriptive group statistics (median (*Mdn*), interquartile range (*IQR*)) and results of Mann–Whitney-U test are visualized.

		SUV_max_		SUV_mean_		ADC_mean_	
		DTC- Negative	DTC- Positive	DTC- Negative	DTC- Positive	DTC- Negative	DTC- Positive
**Right** **femur**	*Mdn* (*IQR*)	2.01 (1.27)	2.06 (1.18)	0.81 (0.46)	0.80 (0.45)	406.33 (334.25)	389.45 (284.17)
	Mann–Whitney-U	*U* = 1880.00, *Z* = −0.61, *p* = 0.54, *r_rb_* = −0.05		*U* = 1940.50, *Z* = −0.33, *p* = 0.75, *r_rb_* = −0.03		*U* = 1950.00, *Z* = −0.28, *p* = 0.78, *r_rb_* = −0.03	
**Os** **sacrum**	*Mdn* (*IQR*)	3.26 (1.23)	3.31 (1.34)	1.54 (0.46)	1.53 (0.51)	508.94 (196.60)	438.55 (233.28)
	Mann–Whitney-U	*U* = 2099.00, *Z* = −0.31, *p* = 0.76, *r_rb_* = 0.03		*U* = 2149.00, *Z* = −0.08, *p* = 0.93, *r_rb_* = −0.01		*U* = 1724.00, *Z* = −1.73, *p* = 0.08, *r_rb_* = −0.15	
**Right os** **ilium**	*Mdn* (*IQR*)	2.85 (1.39)	2.06 (1.18)	1.52 (0.66)	1.44 (0.52)	610.32 (206.80)	622.52 (216.27)
	Mann–Whitney-U	*U* = 1626.00, *Z* = −0.17, *p* = 0.86, *r_rb_* = 0.02		*U* = 2138.50, *Z* = −0.13, *p* = 0.90, *r_rb_* = −0.01		*U* = 2011.00, *Z* = −0.41, *p* = 0.68, *r_rb_* = −0.04	
**L5**	*Mdn* (*IQR*)	3.05 (1.35)	3.21 (1.22)	1.61 (0.67)	1.61 (0.64)	528.70 (268.14)	547.02 (218.11)
	Mann–Whitney-U	*U* = 2005.50, *Z* = −0.62, *p* = 0.54, *r_rb_* = 0.05		*U* = 1949.00, *Z* = −0.88, *p* = 0.38, *r_rb_* = −0.08		*U* = 2071.00, *Z* = −0.13, *p* = 0.89, *r_rb_* = −0.01	
**T7**	*Mdn* (*IQR*)	3.61 (1.52)	3.92 (1.11)	2.00 (0.72)	2.03 (0.56)	461.64 (200.83)	411.32 (258.80)
	Mann–Whitney-U	*U* = 2018.00, *Z* = −0.67, *p* = 0.50, *r_rb_* = 0.06		*U* = 2141.00, *Z* = −0.12, *p* = 0.91, *r_rb_* = −0.01		*U* = 1866.00, *Z* = −1.36, *p* = 0.18, *r_rb_* = −0.12	
**Sternum**	*Mdn* (*IQR*)	1.76 (0.74)	1.75 (0.96)	0.89 (0.41)	0.88 (0.37)	616.46 (232.97)	597.26 (244.70)
	Mann–Whitney-U	*U* = 1990.00, *Z* = −0.80, *p* = 0.43, *r_rb_* = 0.07		*U* = 2122.00, *Z* = −0.21, *p* = 0.84, *r_rb_* = 0.02		*U* = 1950.00, *Z* = −0.58, *p* = 0.56, *r_rb_* = −0.05	

## Data Availability

The datasets used and/or analyzed during the current study are available from the corresponding author upon reasonable request.
